# Prevalence and Molecular Characteristics of Extended-Spectrum β-Lactamase Genes in *Escherichia coli* Isolated from Diarrheic Patients in China

**DOI:** 10.3389/fmicb.2017.00144

**Published:** 2017-02-13

**Authors:** Li Bai, Lili Wang, Xiaorong Yang, Juan Wang, Xin Gan, Wei Wang, Jin Xu, Qian Chen, Ruiting Lan, Séamus Fanning, Fengqin Li

**Affiliations:** ^1^Key Laboratory of Food Safety Risk Assessment, Ministry of Health, China National Center for Food Safety Risk AssessmentBeijing, China; ^2^Institute for Nutrition and Food Hygiene, Beijing Key Laboratory of Diagnostic and Traceability Technologies for Food Poisoning, Beijing Center for Disease Prevention and ControlBeijing, China; ^3^Center for Disease Control and Prevention of Sichuan ProvinceSichuan, China; ^4^College of Veterinary Medicine, Northwest A&F UniversityXianyang, China; ^5^School of Biotechnology and Biomolecular Sciences, University of New South Wales, SydneyNSW, Australia; ^6^UCD-Centre for Food Safety, School of Public Health, Physiotherapy and Sports Science, University College DublinBelfield, Ireland

**Keywords:** *Escherichia coli*, human, extended-spectrum β-lactamase genes, horizontal gene transfer, conjugation, S1 nuclease digestion, China

## Abstract

**Background:** The emergence and spread of antimicrobial resistance has become a major global public health concern. A component element of this is the spread of the plasmid-encoded extended-spectrum b-lactamase (ESBL) genes, conferring resistance to third-generation cephalosporins. The purpose of this study was to investigate the molecular characteristics of ESBL-encoding genes identified in *Escherichia coli* cultured from diarrheic patients in China from 2013 to 2014.

**Materials and Methods:** A total of 51 *E. coli* were confirmed as ESBL producers by double-disk synergy testing of 912 *E. coli* isolates studied. Polymerase chain reaction (PCR) and DNA sequencing were performed to identify the corresponding ESBL genes. Susceptibility testing was tested by the disk diffusion method. Plasmids were typed by PCR-based replicon typing and their sizes were determined by S1-nuclease pulsed-field gel electrophoresis. Multi-locus sequence typing (MLST) and phylogrouping were also performed. Broth mating assays were carried out for all isolates to determine whether the ESBL marker could be transferred by conjugation.

**Results:** Of the 51 ESBL-positive isolates identified, bla_CTX-M_, bla_TEM_, bla_OXA_, and bla_SHV_ were detected in 51, 26, 3, 1 of these isolates, respectively. Sequencing revealed that 7 bla_CTX-M_ subtypes were detected, with bla_CTX-M-14_ being the most common, followed by bla_CTX-M-79_ and bla_CTX-M-28_. Of the 26 TEM-positive isolates identified, all of these were bla_TEM-1_ genotypes. All isolates contained one to three large plasmids and 10 replicon types were detected. Of these, IncFrep (*n* = 50), IncK/B (*n* = 31), IncFIB (*n* = 26), IncB/O (*n* = 14), and IncI1-Ir (*n* = 8) replicon types were the predominating incompatibility groups. Twenty-six isolates demonstrated the ability to transfer their cefotaxime resistance marker at high transfer rates. MLST typing identified 31 sequence types and phylogenetic grouping showed that 12 of the 51 donor strains belonged to phylogroup B2.

**Conclusion:** This study highlights the diversity of the ESBL producing *E. coli* and also the diversity of ESBL genes and plasmids carrying these genes in China, which poses a threat to public health.

## Introduction

Extended-spectrum β-lactamase (ESBL) producing *Escherichia coli* are a frequent cause of community- and hospital-acquired infections and one of the leading causative agents of infections worldwide (Hampton, 2013; Nischal, 2014). *E. coli* can become resistant to extended-spectrum cephalosporins by mutational overproduction of AmpC and/or by expression of acquired ESBLs. The latter emerged in the 1980s as derivatives of TEM (named after the patient Temoneira) and SHV (sulfhydryl reagent variable) enzyme types (Gutkind et al., 2013). The genes encoding these acquired enzymes are associated with plasmids with the potential for horizontal dissemination, and the most widespread ESBL type identified is CTX-M (Gutkind et al., 2013). These ESBL genotypes have spread in a pandemic manner and been associated with outbreaks in hospitals and communities worldwide (Gutkind et al., 2013).

Plasmid-mediated transfer of drug resistance-encoding genes among bacterial species is considered to be one of the most important mechanisms driving the dissemination of multi-drug resistance (MDR). An important element of this MDR dissemination is the transmission of the plasmid-encoded ESBL genes, which confer resistance to third-generation cephalosporins. Infection with ESBL-positive bacteria frequently results from inappropriate use of antimicrobial compounds in human and veterinary medicine a feature that also constitutes a risk factor for selection and dissemination of resistant clones (Gutkind et al., 2013; Hamprecht et al., 2016). Consequently, the treatment options for infections caused by ESBL-producing microorganisms are limited.

ESBL-producing *E. coli* has been reported from China, mostly from extra-intestinal *E. coli* cultured from patient specimens such as bloodstream infections (BSIs), along with intra-abdominal infections (IAIs) and urinary tract infections (UTIs). Some 67.8% of the isolates from BSIs were identified to be positive for the predominant CTX-M enzymes, CTX-M-14 and CTX-M-15 (Wang S. et al., 2016), 68.2% isolates were from UTIs and ICU and which carried the CTX-M-15 gene (Liu et al., 2015) and 69.6% were from IAIs and were also found to be ESBL-producing *E. coli* (Fan et al., 2016). However, there is limited epidemiological information on ESBL-producing *E. coli* from diarrheic patients. In this paper, we report on the isolation and characterization of a collection of ESBL-producing *E. coli* isolated from diarrheic patients in China. The aims of this study were (i) to identify the plasmids carrying ESBL-encoding genes, (ii) to determine the phylogenetic grouping, the multi-locus sequence typing (MLST), and the pathogenic grouping, and (iii) to determine the plasmid replicon types and S1 nuclease-based plasmid profiles.

## Materials and Methods

### Ethics Statement

Fecal samples were acquired with the written consent from all patients. This study was reviewed and approved by the ethics committee of China Center for Disease Prevention and Control, according to the medical research regulations of the Ministry of Health, in China. All of this research work was conducted within China.

### Bacteria and Growth Conditions

Fifty-one isolates including 27 from Beijing, 9 from Guangxi province, 9 from Henan province, and 6 from Sichuan province were confirmed as ESBL producers by the double-disk synergy test from 912 *E. coli* recovered from diarrhea cases. All human samples were collected in urban areas, and each individual was tested only once. The protocols for isolating *E. coli* were described previously (Wang et al., 2013).

Isolates from patients with typical *E. coli* phenotypes were confirmed by the API 20E biochemical gallery (bioMérieux, Beijing, China). Those *E. coli* expressing an ESBL phenotype were selectively enriched for study using Luria Bertani (LB) supplemented with 2 mg/L cefotaxime. All *E. coli* isolates that were recovered were further screened for ESBL production by determination of synergy between 0.25 and 128 mg/L ceftazidime or cefotaxime and 4 mg/L clavulanic acid (CLSI, 2012). Isolates showing a ≥3 twofold concentration decrease in an minimal inhibitory concentration (MIC) for either ceftazidime or cefotaxime tested in combination with clavulanic acid versus the MIC of the agent when tested alone were considered as ESBL-producing (for example, ceftazidime MIC = 8 mg/L; ceftazidime–clavulanic acid MIC = 1 mg/L; CLSI, 2012). *E. coli* ATCC 25922 and *Klebsiella pneumoniae* ATCC 700603 were used as quality control microorganisms in antimicrobial susceptibility tests (CLSI, 2012). All confirmed isolates were maintained in brain heart infusion broth containing 50% [v/v] glycerol in a -70°C freezer for subsequent genotypic and phenotypic characterization.

### Antimicrobial Susceptibility Testing

Antimicrobial susceptibility of all *E. coli* isolates was determined using the agar dilution method and interpreted according to the Clinical and Laboratory Standards Institute guidelines (CLSI, 2012) and the European Committee on Antimicrobial Susceptibility Testing (EUCAST-2012)^1^. The following antimicrobial compounds were assessed: ampicillin (AMP, 1–128 mg/L), cefazolin (CZO, 0.5–64 mg/L), cefotaxime (CTX, 0.015–128 mg/L), cefotaxime–clavulanic acid, ceftazidime (CAZ, 0.03–64 mg/L), ceftazidime–clavulanic acid, chloramphenicol (CHL, 1–128 mg/L), ciprofloxacin (CIP, 0.015–512 mg/L), gentamicin (GEN, 0.125–64 mg/L), imipenem (IMP, 0.03–16 mg/L), tetracycline (TET, 0.25–64 mg/L), tigecycline (TGC, 0.015–32 mg/L), and trimethoprim–sulfamethoxazole (SXT, 0.06/1.19-16/304 mg/L). MDR was defined as resistance to three or more different classes of agent (Wang et al., 2013).

### PCR Amplification and DNA Sequence Analysis of ESBLs

The following ESBLs resistance determinants were investigated by polymerase chain reaction (PCR): *bla*_carB_, *bla*_CMY -2_, *bla*_CTX-M_, *bla*_OXA_, *bla*_SHV_, *bla*_TEM_ (Xu et al., 2005; Karczmarczyk et al., 2011). All PCR products amplified from *bla*_CTX_ and *bla*_TEM_ genes were commercially sequenced (Takara Biotechnology Cooperation, Dalian, China) and subsequently analyzed using the BLAST program^2^.

### Plasmid Characterization: S1 Nuclease Digestion and PCR-Based Plasmid Replicon Typing

S1-nuclease (Promega, Madison, WI, USA) digestion as well as pulsed-field gel electrophoresis (PFGE) analysis was performed for all 51 isolates. Briefly, the procedure included a lysis step of the bacterial cells embedded in agarose plugs followed by digestion with 8 U S1 nuclease at 37°C for 30 min. Finally, each plasmid sample was resolved by PFGE in a Chef-Mapper^®^ XA System (Bio-Rad, USA) at 14°C, with a switch time between 1 and 12 s, at 6 V/cm on a 120° angle in 0.5× tris-boric acid-ETDA (TBE) buffer for 18 h. Each DNA band identified was considered as a unit length of linear plasmid. The approximate sizes of plasmids were determined by comparing profiles with *Xba*I-digested DNA from *Salmonella* serotype Braenderup H9812. Plasmids were assigned to incompatibility groups on the basis of the presence of specific replicon sequences identified by PCR using the primers previously designed and the corresponding amplification protocols described (Wang et al., 2013).

### Conjugation-Based Mating Experiments and Verification

Conjugation experiments were performed using a broth mating protocol to determine if plasmids coding for CTX-M and TEM enzymes could be transferred (Barton et al., 1995). Fifty-one donors were used for mating with a sodium azide-resistant *E. coli* J53 as previously described (Wang et al., 2013). Transconjugants were selected on LB agar plates containing sodium azide (100 mg/L) and cefotaxime (2 mg/L). PCR amplification, antimicrobial susceptibility testing, plasmid replicon typing, and S1-PFGE were performed for all the transconjugations to determine the presence of ESBL genes, antibiotic phenotypes and incompatibility groups, respectively (Wang et al., 2013).

### Phylogenetic Grouping

Phylogenetic groups were determined for each *E. coli* isolate using an established multiplex PCR targeting *chuA* (279 bp), *yjaA* (211 bp), and *tspE4* (152 bp) according to the protocol of Clermont et al. (2000). The method was previously developed to classify *E. coli* into four phylogenetic groups designated A, B1, B2, and D.

### Multi-locus Sequence Typing of Isolates

MLST analysis was conducted by sequencing fragments of seven housekeeping genes (*adk, fumC, gyrB, icdF, mdh, purE, recA*) and sequence types (STs) were assigned using the *E. coli* MLST website (Wirth et al., 2006). eBURST was performed for MLST analysis.

### Detection of Virulence Genes of Diarrheagenic *E. coli*

Five distinct classes of diarrheagenic *E. coli* (DEC) are recognized as being associated with diarrheal disease: enteropathogenic *E. coli* (EPEC), Shiga toxin-producing *E. coli* (STEC), enteroaggregative *E. coli* (EAEC), enteroinvasive *E. coli* (EIEC), and enterotoxigenic *E. coli* (ETEC). PCR was used to distinguish these *E. coli* pathotypes by amplification of the following gene targets: typical EPEC (*eae* and *bfp*), atypical EPEC (*eae* or *bfp*), STEC (*eae* and *stx1* and/or *stx2*), ETEC (*elt* and/or *estIa* or *estIb*), EIEC (*invE* and *ipaH*), and EAEC (*aggR* and/or *aatA* or *aaiC*) (Imuta et al., 2016; Tian et al., 2016).

## Results

### Antimicrobial Susceptibility Testing of 51 ESBLs Isolates

**FIGURE 1:**
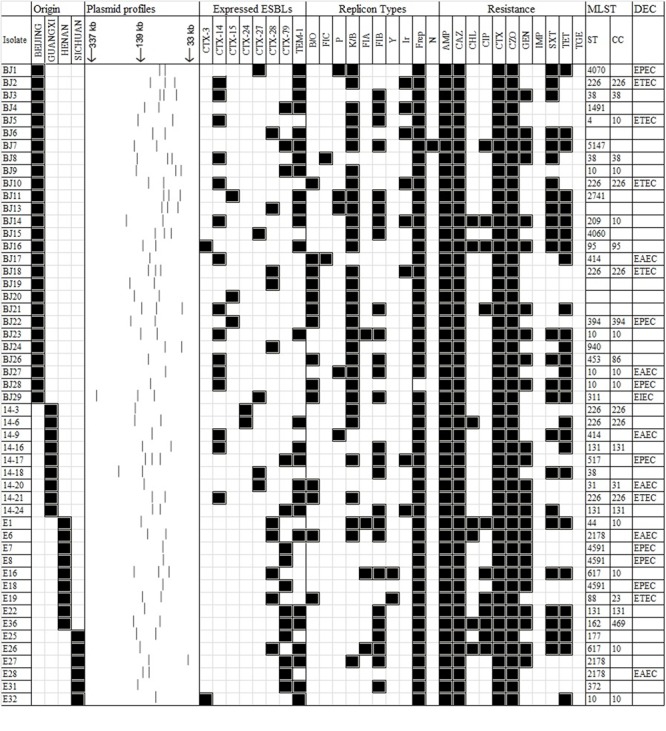
**A heat-map summary of the sources; a schematic showing the S1 nuclease plasmid profile; ESBL markers identified by PCR and sequencing, the resistance profile, the corresponding Inc plasmid type(s), MLST and DEC type for all 51 ESBL-positive *E. coli* previously reported from humans in China.** Black squares shown indicate a feature present in that isolate denoting its original source the ESBL marker(s) detected, its corresponding antibiogram profile and the Inc types detected. White squares denote features that are lacking in the corresponding bacterial isolate. Antimicrobial compounds used are abbreviated as follows: AMP, ampicillin; CAZ, ceftazidime; CHL, chloramphenicol; CIP, ciprofloxacin; CTX, cefotaxime; CZO, cefazolin; GEN, gentamicin; IMP, imipenem; SXT, trimethoprim/sulfamethoxazole; TET, tetracycline, TGC, tigecycline.

All 51 ESBL-producing *E. coli* isolates were found to be resistant to ampicillin, cefazolin, cefotaxime, ceftazidime, and susceptible to imipenem and tigecycline. Resistance to tetracycline (27/51, 53.0%) was most common, followed by resistance to trimethoprim–sulfamethoxazole and gentamicin (49.0%, 25/51). All *E. coli* isolates expressed a MDR phenotype (being resistant to at least three different classes of antimicrobial compounds; **Figure 1**).

### Identification of β-Lactamase Resistance-Encoding Genes

Of the 51 isolates, *bla*_CTX-M_, *bla*_TEM_, *bla*_OXA_, and *bla*_SHV_ were detected in 51, 26, 3, and 1 isolates, respectively. Sequencing revealed seven *bla*_CTX-M_ subtypes: *bla*_CTX-M-14_ (*n* = 15), *bla*_CTX-M-79_ (*n* = 14), *bla*_CTX-M-28_ (*n* = 10), *bla*_CTX-M-27_ (*n* = 5), *bla*_CTX-M-15_ (*n* = 3), *bla*_CTX-M-24_ (*n* = 2), and *bla*_CTX-M-3_ (*n* = 2). Of the 26 TEM-positive strains, all were *bla*_TEM-1_.

### S1 Nuclease Digestion and PCR-Based Plasmid Replicon Typing

S1 nuclease-based plasmid analysis showed that all 51 ESBL-positive isolates contained detectable large plasmids; most possessed two plasmids (*n* = 24, 47.1%) and some (*n* = 12, 23.5%) had three plasmids. All of the remaining *E. coli* isolates harbored a single plasmid. Heterogeneity among the profiles was a common feature noted (**Figure 1**). Eighteen plasmid replicons were detected by qualitative PCR-based plasmid replicon typing (PBRT) among those 51 isolates carrying large plasmids. PBRT typing identified 10 of 18 replicons. Interestingly, IncA/C, IncT, IncW, IncFIIA, IncX IncHI1, IncHI2, or IncL/M types were not be detected by PCR analysis in our collection. IncFrep (*n* = 50), IncK/B (*n* = 31), IncFIB (*n* = 26), IncB/O (*n* = 14), and IncI1-Ir (*n* = 8) replicon types were the predominated types. Several isolates were positive for more than one replicon type (*n* = 46). A summary of these features along with the corresponding antimicrobial resistance profiles for all 51 isolates is shown as a heat-map in **Figure 1**.

### Conjugational Transfer of Resistance Carried by the 26 *E. coli* Isolates

**FIGURE 2:**
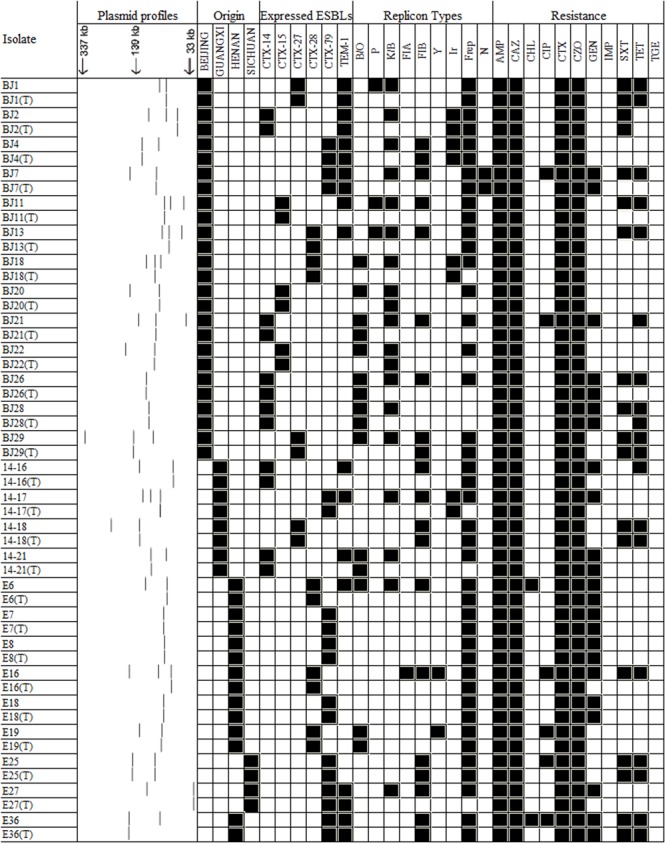
**A heat-map showing the comparison of ESBL-positive *E. coli* donors and the resultant transconjugants, characterized on the basis of their plasmid profiles; ESBL-markers identified by PCR; antimicrobial resistance profile; and plasmid replicon type(s).** Antimicrobial compounds are abbreviated as described in the legend to **Figure 1**.

Each of the 51 *E. coli* isolates expressing an ESBL phenotype, was tested for its ability to transfer the phenotype, by conjugation under laboratory conditions. Twenty-six isolates transferred the cefotaxime resistance marker to a susceptible *E. coli* recipient with frequencies ranging from 3.8 × 10^-2^ to 4.2 × 10^-1^ transconjugants *per* donor cell (**Figure 2**). Following conjugation the antimicrobial susceptibility profiles of all 26 transconjugants was determined by the disk diffusion method. In addition to cefotaxime resistance, resistance to several non-β-lactam-based antimicrobial compounds such as gentamicin, tetracycline, and trimethoprim–sulfamethoxazole were also transferred to the recipient, suggesting that these might be located on the same plasmid. For transconjugants, the most commonly detected *bla*_CTX-M_-encoding genes included *bla*_CTX-M-79_ followed by *bla*_CTX-M-14_ and *bla*_CTX-M-28_. It should be noted that nearly half of those genes both in the *bla*_CTX-M-1_ group and the *bla*_CTX-M-9_ group were transferable to the recipient bacterium, under laboratory conditions. S1-PFGE analysis showed that the donor isolates carried multiple plasmids with sizes ranging from 34- to 294-kb.

### Phylogenetic Grouping and MLST

In all 51 isolates tested, 12 were assigned to B2 and 10 assigned to group D. Twenty isolates were assigned to group A and 9 were assigned to group B1 (**Figure 1**). MLST sub-typing identified 27 types along with 5 new STs not previously registered in the *E. coli* MLST database [including the isolates BJ6 (58-53-12-58-24-01-42), BJ13 (43-41-15-08-11-08-06), BJ19 (261-160-02-63-55-04), BJ20 (261-160-02-63-55-04), BJ20 (56-11-04-10-07-08-06)]. The most prevalent ST was ST266, followed by ST10, ST38, ST131, ST2178, and ST4591. Most of these strains belonged to clonal complexes (cc) of ST10cc, ST266cc, ST131cc, and ST38cc in **Figure 1**.

### Occurrence of Pathogens

PCR screening of the typical virulence genes, revealed that 20 of the 51 ESBL-producing study isolates can be assigned to a pathotype with seven EPEC, six ETEC, six EAEC, and one ETEC isolate, being noted (**Figure 1**).

## Discussion

ESBL-producing *E. coli* are important causative agents of food-borne infections worldwide (Nischal, 2014). In China, 60.7, 88.8, and 57.1% ESBL-positive *E. coli* were detected from isolates sampled from chicken farms in Henan province (Yuan et al., 2009), healthy broilers in Shandong province (Li et al., 2016), and in piglets (Zhang et al., 2015) separately which in turn may transmit to the human population causing food-borne infections (Leverstein-van Hall et al., 2011). Therefore, tracking the transmission routes of food-borne *E. coli* in community settings is a necessary step required as efforts are made to limit its continuous spread. In China, there is limited information available to describe the nature of ESBL-positive bacteria cultured from patients with diarrhea (Liu et al., 2015; Fan et al., 2016).

In this study, the observed a prevalence of 5.6% (51/912) that was similar to data published previously (Valenza et al., 2014; Hamprecht et al., 2016). Plasmids confer positively selectable phenotypes including antimicrobial resistance genes among others (Andersson and Hughes, 2010). These mobile genetic elements represent an important pool of adaptive and transferable genetic information (with large plasmids defined as being >30-kb in size; Andersson and Hughes, 2010).

Resistance to third-generation cephalosporins was most commonly associated by ESBLs of the CTX-M-1 group in the present study (54.9%), followed by ESBLs of the CTX-M-9 group (45.1%). When comparing these data with other studies from China investigating only *E. coli*, the prevalence of the CTX-M-1 group (55.7%) and CTX-M-9 group (43.3%) among *E. coli* ESBLs was similar to those values reported in an earlier study detected from BSIs (Wang S. et al., 2016), but different compared with the prevalences in UK, the Netherlands, and Germany in which CTX-M-1 was the dominant group compared to CTX-M-9 not only from human isolates but also in those cultured from food-producing animals (Wu et al., 2013; Hamprecht et al., 2016). Genotypes corresponding to several CTX-M enzymes were identified, the majority of which were found to be of the *bla*_CTX-M-14_ sub-type, compared with other CTX-M types (such as *bla*_CTX-M-15_) identified in hospitals (Liu et al., 2015; Wang L.H. et al., 2016) which differed from data reported from a single survey of retail foods (which identified *bla*_CTX-M-55_) in China (Xu et al., 2014).

As TEM-1 is the most common plasmid-mediated β-lactamase identified in enteric Gram-negative bacilli, high rates of ampicillin-resistant, corresponding with *bla*_TEM-1_ positive isolates are to be expected. In the current study, the *bla*_TEM-1_ gene was also identified in six transconjugants (**Figure 2**). Particular plasmid incompatibility groups are more frequently encountered among *E. coli* and these are thought to play a major role in the dissemination of specific resistance genes. Interestingly, *bla*_CTX-M_ genes were found to be located predominantly on IncFrep or IncK/B types and conjugation experiments showed that *bla*_CTX-M_ from 26 isolates were transferable, with IncFrep and IncFIB being the most prevalent replicon types, a feature that differed from data reported previously (Wang et al., 2013; Xie et al., 2016). Most of the transferable isolates belonged to the phylogenetic group A followed by groups-B2, -B1, and -D. Since virulent organisms causing infections are commonly associated with phylogroup B2, and to a lesser extent group D, most of the isolates in this study belonged to phylogroups-B1 and -A, which are regarded as commensal bacteria (Wirth et al., 2006). Based on data obtained from the *in vitro* conjugation experiments, some 51% of the isolates that were capable of transferring one or more plasmids were phylogrouped as commensal isolates. This finding is consistent with previous reports showing that commensal *E. coli* in food-producing animals were likely to be a reservoir of ESBL-encoding genes and these bacteria played a role in the dissemination of such mobile elements (Smet et al., 2010). To our surprise, nearly half of the isolates are DEC. It would appear that the CTX-M resistance mechanism has spread to several types of *E. coli*. Moreover EAEC O25:H4/CG131 is thought to have evolved to harbor CTX-M plasmids (Imuta et al., 2016).

The diversity of MLST profiles identified suggested that the *bla*_CTX-M_ genes were acquired as a result of horizontal transfer from existing resistant organisms, rather than clonal expansion of specific resistant strains (Adler et al., 2016). ST10 clone complex was the most prevalent STcc detected in our study and which are associated with some are pathogenic *E. coli* types such as ETEC, EAEC, EPEC. Also this ST10cc was reported earlier in Europe (Day et al., 2016) and was associated with the production of different CTX-M enzymes. Further it has been observed that the latter can harbor the recently reported *mcr-1* gene (Bernasconi et al., 2016). ST266 was also detected and this is a ST type that had not previously been reported, therefore it may represent a new MLST type identified in China. Most of the ST266 types observed were identified as ETEC isolates, which is a major cause of acute secretory diarrhea and often referred to as the cause of travelers’ diarrhea, being one of the major causes of diarrheal disease in children from developing countries (Joffre et al., 2016). ST131 is a globally spread clonal group of extraintestinal pathogenic *E. coli* (ExPEC), comprising different sub-lineages with the ability to acquire and spread antibiotic resistance and virulence genes *via* plasmids. It is seldom detected in companion and food-producing animals (Lanza et al., 2014; Day et al., 2016; Imuta et al., 2016; Wang L.H. et al., 2016). In this study, only three ST131-B2 isolates associated with the production of different CTX-M enzymes (including the genotypes *bla*_CTX-M-14_ or *bla*_CTX-M-79_ with *bla*_TEM-1_) were identified in contrast to earlier reports (Lanza et al., 2014). This ST poses a major threat to public health because of its global distribution. Identification of ST131 isolates that produce CTX-M has important implications for the future treatment of community-associated infections. The potential widespread dissemination of ST131 with its distinctive combination of resistance and virulence requires further investigation.

## Conclusion

The *bla*_CTX-M_ positive *E. coli* characterized in this study pose a serious challenge for the treatment of human infections. These isolates were found to be not only resistant to cephalosporins but to other classes of antimicrobial compound. Continuous investigation and surveillance will extend our understanding of the transmission dynamics and the evolution of these isolates.

## Author Contributions

LB, RL, SF, and FL designed the experiments. LB, SF, and FL wrote the paper. LB, LW, and JW did the experiments and analyzed the data. XY, XG, WW, and QC collected the strains and analyzed the data. JX discussed the results.

## Conflict of Interest Statement

The authors declare that the research was conducted in the absence of any commercial or financial relationships that could be construed as a potential conflict of interest.

## Footnotes

1 http://www.eucast.org

2 http://www.ncbi.nlm.nih.gov/BLAST/
